# Microbial Degradation of Azo Dyes: Approaches and Prospects for a Hazard-Free Conversion by Microorganisms

**DOI:** 10.3390/ijerph19084740

**Published:** 2022-04-14

**Authors:** Anna Christina R. Ngo, Dirk Tischler

**Affiliations:** Microbial Biotechnology, Ruhr University Bochum, Universitätsstr. 150, 44780 Bochum, Germany; anna.ngo@rub.de

**Keywords:** azo dye degradation, decolorization, bioremediation, immobilization, *p*-phenylenediamine, xenobiotics

## Abstract

Azo dyes have become a staple in various industries, as colors play an important role in consumer choices. However, these dyes pose various health and environmental risks. Although different wastewater treatments are available, the search for more eco-friendly options persists. Bioremediation utilizing microorganisms has been of great interest to researchers and industries, as the transition toward greener solutions has become more in demand through the years. This review tackles the health and environmental repercussions of azo dyes and its metabolites, available biological approaches to eliminate such dyes from the environment with a focus on the use of different microorganisms, enzymes that are involved in the degradation of azo dyes, and recent trends that could be applied for the treatment of azo dyes.

## 1. Introduction

Colors play an important role to different industries and in consumer choices. It also contributes to the aesthetic quality of a product, which drives consumers to purchase and therefore contributes to economic growth. The different colors and hues that we see are often derived from the dyes that were used. Natural dyes are the safer and more eco-friendly option compared to synthetic dyes [1]. They also pose other advantages such as having antimicrobial properties and offering protection from UV light [2,3,4,5].

Although natural dyes are the safer and the more environmentally friendly option to use, they are quite costly, more tedious to apply, and more difficult to procure. Tyrian purple, for example, is a natural dye from the mucus of *Murex* sp. snails that retails for about €2000 per gram [6,7]. Therefore, the use of natural dyes has been deemed to be impractical for many commercial applications. Moreover, natural sources of dyes usually contain only about 2% of actual coloring material, which means that uneconomic amounts of raw material might be needed to obtain the desired shades and hues. This drives up its cost and is thus undesirable for mass production use. It is nearly impossible to reproduce the same shade from batch to batch and fastness properties are rather poor, which make it difficult to apply on textiles [1].

As an alternative, synthetic dyes such as azo dyes have become the primary coloring agent in industries such as the food, cosmetics, and textile industries as trace amounts of dyeing material already produce an intense color [8,9,10]. Azo dyes take account for most of the synthetic dyes used as they are easy to synthesize, thus cost-efficient to produce, and generate a variety of colors as there are about 10,000 different azo dyes available [10,11,12]. Since azo dyes are synthesized chemically, careful downstream treatments are needed to ensure safety in its usage and disposal [13]. It is important to consider that only 10% of the dye is transferred into the material permanently, and the majority goes into wastewater as effluents [13]. At the same time, trace amounts of dye can lead to severe environmental and health hazards as some azo dyes are toxic, carcinogenic, and mutagenic [14,15]. The presence of these synthetic dyes can also hamper various biological activities [16]. Therefore, it is important to implement tight regulations for their treatment and disposal.

Several physical and chemical procedures are available for the downstream treatment and waste handling of azo dye containing effluents [17,18,19]. Quite often, these approaches are met with skepticism, as these have major downsides such as the ecologically questionable waste disposal for filters and charcoals, generation of toxic intermediates, production of sludge, and the high costs of equipment [19,20]. Thus, finding a more environmentally friendly approach is essential. Here, biological methods can be a more promising solution. This review tackles the treatment methods available for azo dyes especially on the biological context (primarily bacteria) with emphasis on the approach of whole-cell biocatalysis and/or enzymatic degradation. Physical parameters that affect dye decolorization and degradation, such as pH and temperature, are not within the scope of this review.

## 2. Impacts of Azo Dyes on Human Health and the Environment

**Figure 1:**
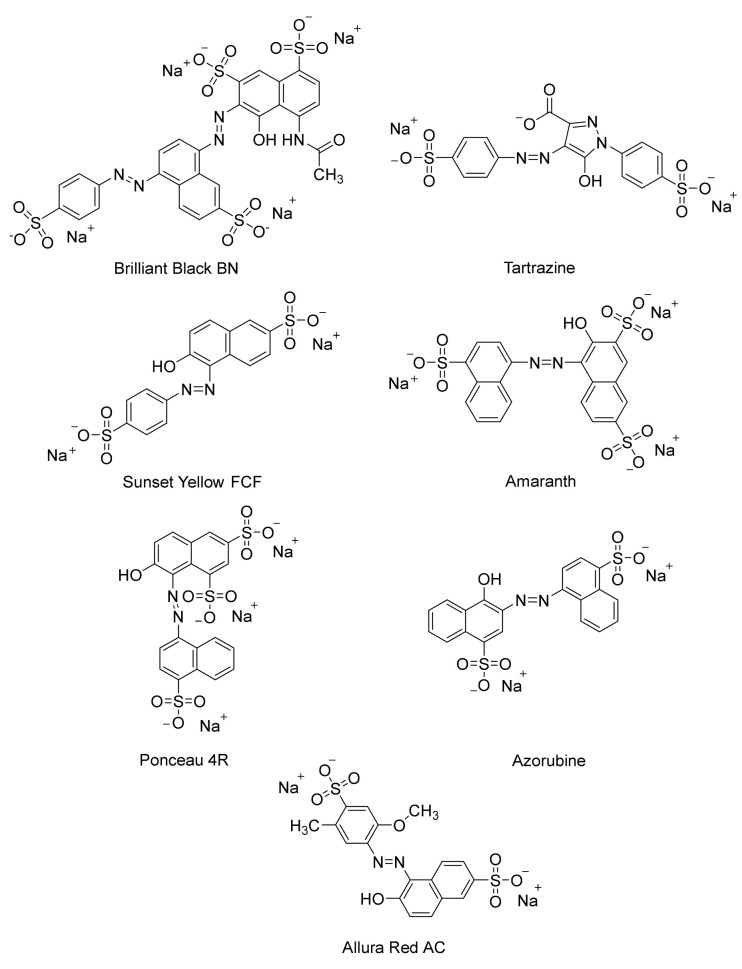
Commonly used azo dyes in the food industry.

Azo dyes are characterized by the presence of an azo bond (-N = N-) between two or more aromatic rings [21,22]. The versatility of azo dyes renders them very appealing for various industries such as food and textiles. However, the xenobiotic nature of these dyes calls for proper evaluation of their harmful effects. In the food industry, the use of azo dyes should be critically assessed as they are often used as colorants for sweets and desserts with children as target consumers [23]. Although there are regulations on which dyes may be used or not, these are different by country, which adds to the difficulty of standardizing protocols for the usage of dyes [23]. There are thousands of azo dyes used in various industries. Some of the better-known azo dyes used in the food industry are Brilliant Black BN, Tartrazine, Sunset Yellow FCF, Amaranth, Azorubine, Ponceau 4R, and Allura Red AC [23] (Figure 1).

The toxicity of some of these azo dyes can be attributed to the reduction of the azo bond, which produces aromatic amines [24,25,26]. When ingested orally, the dye reaches the gastrointestinal tract, and the intestinal microflora or mammalian azoreductases cleave the azo bond [25,27,28]. The aromatic amines, which are often the final product of azo dye reduction, are subsequently hydroxylated or acetylated, and this adds to the mutagenicity and carcinogenicity of these compounds. The intake of azo dyes can also increase the risk of human bladder cancer, splenic sarcomas, hepatocarcinomas, and nuclear anomalies [25]. They can also cause allergies, dermatitis, and even DNA damage that results in the formation of malignant tumors [29,30]. Methyl Yellow, which is now banned in different countries, was formerly used for dyeing butter, and it was found to cause liver cancer in rats after two to three months of exposure [31,32]. Another example is the dye Amaranth, which was shown to induce DNA damage in the colon epithelium of mice [33,34,35], while Brilliant Black BN has shown genotoxicity in human lymphocytes based on in vitro experiments [36]. Sunset Yellow FCF can cause DNA damage and has shown to have toxic effects on the reproductive and neurobehavioral system of tested rodents and chick embryos [37,38]. Tartrazine was shown to bind to albumin, induce neurotoxicity, impair mental functions, and promote various reactions such as angioedema, nasal congestion, itchy skin, and hives [23]. In the cosmetic and textile industries, 4-aminobenzene or Aniline Yellow, a dye used for printers and as a precursor of other dyes, has been shown to cause high hepatocarcinogenicity and induce tumors to rats [39]. Sudan III, used for coloring non-polar substances such as acrylic emulsions, was also reported as a carcinogen [40].

Aside from health issues, another significant problem of these azo dyes is their presence in the environment. Trace amounts of azo dyes in watercourses can cause aesthetic pollution. It also leads to different chain reactions. Poor sunlight penetration into the water decreases the photosynthetic activity from waterborne organisms, leading to a reduction in dissolved O_2_ concentration, and it can as a consequence cause acute effects to the aquatic community [26]. The dissolved O_2_ concentration primarily restricts the growth and development of plants. These dyes can also affect germination rates and even inhibit the elongation of shoots and root seedlings [22,26]. As plants have a significant role in ecology such as serving as a habitat for organisms, providing organic matters that contribute to soil fertility, and keeping the soil from erosion, all these services are threatened as dye-containing effluents, and other xenobiotics continue to be discharged to the environment [22]. Moreover, the presence of these pollutants negatively affects the chlorophyll content of plants, as they trigger the production of chlorophyllase and abscissic acid, both of which can lead to chlorophyll degradation [26,41]. Aquatic organisms such as fishes are affected by the water quality of their environment. The presence of azo dyes and other toxic compounds impair the growth of several fishes—showing reduced growth and affecting the muscles, gills, liver, and intestines [26]. The edible freshwater fingerling, *Catla catla*, has shown histopathological alterations in gills such as hyperplasia and degenerated central axis when treated with Reactive Red 120 [42]. It was also shown that the exposure of embryo larval fathead minnows, *Pimephales promelas*, to the azo dyes Disperse Yellow 7 and Sudan Red G decreased the survival of larval fish with LC_50_ values of 25.4 µg/L and 16.7 µg/L, respectively [43]. These examples strikingly show how hazardous azo dyes can be to all areas of nature if not properly treated.

## 3. Impact of Azo Dye Metabolites

As mentioned earlier, not all azo dyes are harmful. However, dyes that were not found to be harmful still pose a threat, since oxidative and reductive metabolism could lead to the formation of toxic aromatic amines. Different studies have identified compounds derived from azo dye metabolism such as benzidine, *p*-phenylenediamine, aniline, and toluene, which show carcinogenic and mutagenic properties [24,25,27,31,39]. 

Benzidine is a building block of azo dyes such as Congo Red, Direct Black 38, and Direct Red 39. Earlier studies have shown that it can induce tumor cells on different body parts such as the gastrointestinal tract, pancreas, liver, and gallbladder [39]. Benzidine and its congeners, such as 3′3-dimethylbenzidine and 3′3′-dichlorobenzidine, were shown to induce carcinoma and tumors. 3′,3′,-5,-5′ Tetramethylbenzidine was the only congener of benzidine that was not found to be carcinogenic [39]. 

Meanwhile, *p*-phenylenediamine is another compound that is used as a henna substitute, for the manufacturing of certain polymers in different industries, as a developing agent for films, and as a major component for hair dyes. Initial investigations on *p*-phenylenediamine showed contrasting results. On the one hand, it was shown to be carcinogenic via an Ames test. On the other hand, it was shown that it did not pose any carcinogenic potential to F344 rats regardless of the sex and the exposure time [44]. It was also shown to not affect pregnant rats, and a multigenerational reproduction study also showed no negative effects [44,45]. However, it was discovered that *p*-phenylenediamine only becomes mutagenic after oxidation [46]. This was corroborated when *p*-phenylenediamine, in the presence of microsomal fraction and after H_2_O_2_ oxidation, became mutagenic to a *Salmonella typhimurium* tester strain TA1538 [47]. This poses a problem as most permanent hair dyes need to be oxidized upon usage to exert their dyeing properties. It was shown that upon absorption, *p*-phenylenediamine can lead to the formation of tumors in the liver, kidney, urinary bladder, and thyroid gland of rats [48,49]. It was also found that it can increase the expression of p53 proteins, thereby suggesting an increase in apoptosis and affecting cell viability [49]. A correlation was also observed on the usage of hair dyes and Non-Hodgkin lymphoma and cancer [50].

Other monocyclic aromatic amines such as *p*-nitroaniline, *o*-toluidine (2-methylaniline), 2-nitro-*p*-phenylenediamine, and *o*-phenylenediamine were also shown to have carcinogenic and mutagenic properties [39]. These compounds are all highly relevant toxins, as occupational exposure to aromatic amines can explain 25% of bladder cancer cases [51].

## 4. Physical and Chemical Treatment of Dyes

As azo dyes pose different risks and hazards to health and the environment, it is necessary to find ways to treat these dyes. Various physical and chemical methods have been explored [18,29,52]. Physical methods include techniques such as adsorption and filtration [8]. Adsorption uses materials such as activated carbon that can accumulate compounds to be removed from wastewater at their surface [53,54,55]. Although activated carbon is effective and has been the primary option for this kind of treatment, it is not often used due to its high cost. Alternatives have been explored, such as peat, banana peels, clay, corn cob, maize, and wheat straw [55,56,57,58]. However, there are some drawbacks due to the problematic waste disposal of these cheaper alternatives. Another frequently used physical method is filtration. It often involves the use of membranes to remove suspended solids and other unwanted materials from water [19]. Although effective, it likewise has some drawbacks such as similarly high costs for investment and of materials, deterioration of the membrane or membrane fouling, production of potentially toxic sludge, and again problematic waste disposal [19,22].

Chemical treatment involves the use of different chemicals or techniques such as coagulation–flocculation, Fenton’s reagent, ozone, and electrochemical methods [22]. Coagulation–flocculation followed by sedimentation are processes used in conventional wastewater treatment facilities [59,60]. Coagulation involves the use of coagulants to neutralize suspended solids (often with an opposite charge to the coagulant). Once neutralized, the suspended solids can collide and form microflocs. During flocculation, these microflocs can form macroflocs and sediment, upon which they can be removed from water using gravity. Furthermore, the Fenton reaction allows the generation of hydroxyl radicals using Fenton reagent (H_2_O_2_ and Fe^2+^ ions), which can destroy toxic pollutants in wastewater [61]. Although the overall process is cheap, it can lead to a high sludge production as secondary waste. Ozonation uses reactive ozone (O_3_) to oxidize and disassemble preferentially the chromophores of dyes, but its unstable nature makes it undesirable for wastewater treatment [62]. 

These physical and chemical treatments have been used traditionally for wastewater treatment. However, as mentioned earlier, these approaches can pose several drawbacks. Furthermore, dyes may be resistant or recalcitrant to these available treatments [63]. Therefore, it is necessary to find safer, more eco-friendly ways that can handle these synthetic dyes, especially azo dyes.

## 5. Biological Treatment of Dyes

Microorganisms are ubiquitous. Their ability to withstand harsh conditions and to thrive even in the most polluted environment make them a melting pot for the study of interesting enzymes and metabolites that can be harnessed to potentially solve environmental problems. The use of microorganisms as an alternative to physicochemical treatments is called bioremediation [64,65]. Bioremediation has sparked serious interest in the past decades, and microorganisms have been well-documented to destroy polyaromatic hydrocarbons [66,67,68], to transform heavy metals to less harmful or immobilized forms [69,70], to degrade pesticides [71,72], and to reduce or mineralize azo dyes [73,74]. 

For dye degradation, the use of microorganisms has several advantages. In a comparably cost-efficient manner, the dyes are decomposed to a vast degree whilst less water is required and less sludge produced. The mechanisms of how microorganisms can decolorize (i.e., reduce azo bonds to aromatic amines) and degrade (i.e., break down azo dyes into small molecules leading to H_2_O, CO_2_, and mineral by-products) azo dyes have been a subject of interest in different studies [75,76,77,78,79,80]. Microbiological dye removal or even degradation can be achieved by means of various ways such as via adsorption, via the production of enzymes that can attack the dyes, and even via the combination of both [81,82]. Adsorption occurs through ion exchange as the microbial cell wall has hydroxy and carboxy groups that can serve as binding sites for the dyes to adhere to [83,84]. Adsorption can be achieved by either live cells or dead cell biomass [85,86]. It has several disadvantages: azo dyes cannot be transformed into non-toxic forms, thus making waste disposal similarly problematic as in classical physical methods. As opposed to adsorption, the degradation of azo dyes has been of significant interest as ideally, the dyes can be completely degraded by the microbial enzymes. Microorganisms capable of decolorizing and degrading dyes include filamentous fungi [87,88], yeasts [89,90], algae [91,92], and bacteria [93,94,95]. 

### 5.1. Biological Treatment of Dyes Using Filamentous Fungi

Filamentous fungi are versatile microorganisms that can produce intracellular and extracellular enzymes able to degrade a variety of xenobiotics. They can be isolated everywhere such as in soil and even from waste organic materials. The mechanisms on how filamentous fungi decolorize and degrade azo dyes rely upon the combined activities of the biosorption process and the extracellular enzymes produced [80,96,97]. Moreover, investigations on filamentous fungi as a biosorbent for dye-containing effluents have been performed on both living and dead biomass setups.

The dead biomass of fungi has been of significant interest, as it was shown to take up higher concentration of dyes than the living biomass. The use of dead cells often relies on physicochemical interactions such as adsorption, deposition, and ion exchange to serve as a biosorbent for the treatment of dyes. In this process, dye molecules adhere to the fungal mycelia via ion exchange [80]. The heteropolysaccharides present in the cell wall of fungi such as chitin, chitosan, glucan, lipids, and phospholipids serve as binding sites with functional groups such as carboxy, hydroxy, and phosphoryl groups that help facilitate the biosorption process [80]. It was shown that dead fungal cells of *Aspergillus niger* can take up the dyes Basic Blue 9, Acid Blue 29, and Reactive Brilliant Red [96,98,99]. The integrity of the cell wall also plays an important role, as disrupted cells showed less efficiency in adsorbing dye solutions after 24 h than intact ones [99,100]. Filamentous fungi also serve as a powerhouse source for different enzymes that can convert various dyes. Some of the known fungal enzymes involved in dye degradation are laccases, lignin peroxidases, and manganese peroxidases (Table 1).

Several studies have compared these two approaches for fungal biomass application for dye treatments. It was shown that living and dead cells were equally effective for dye color removal [107]. Meanwhile, a wide screening across different kinds of microorganisms showed that dead forms had better decolorization rates for Reactive Black 5 and Reactive Blue 19 [108]. A study of Przytas et al., compared the efficiency of immobilized fungi, namely *Pleurotus ostreatus* BWPH, *Gleophyllym odoratum* DCa, and *Polyporus picipes*—in living and in autoclaved form, and corroborated previous findings where it was shown that the decolorization rates of the used dyes were higher in dead fungal biomass [109]. 

Both approaches for fungal biomass use have advantages and disadvantages. As mentioned earlier, living cells can have a variety of different mechanisms for dye decolorization and degradation. However, this entails optimizing operating conditions such as pH, moisture, temperature, nutrient supply, and culture maintenance, as all can affect the ability of fungi to secrete enzymes. On the other hand, dead biomass seems to be an effective biosorbent, but just like any (physical) adsorbent, the question of waste disposal will always remain. This is especially true for the then dye-enriched adsorbent. The fungal biomass probably degrades fast, and the previously adsorbed dye persists at the disposal site and might be exposed to weathering.

### 5.2. Biological Treatment of Dyes Using Yeasts

Yeasts are not widely used in dye decolorization and have not been as extensively studied as bacteria and filamentous fungi. However, yeasts hold potential in this field especially because they present a biotechnological advantage as they are fast-growers and can also thrive in harsh conditions. Yeasts typically remove dyes by the process of biosorption. The dyes adhere to cell peripheries and eventually enter into the cell based on interactions made by the functional groups present on the cell surface through electrostatic interactions, ion exchange, or ion chelation. In a screening by Yu and Wen (2005), Reactive Brilliant Red K-2BP was removed through biosorption by *Saccharomyces cerevisae*, *Saccharomyces uvarum*, *Saccharomyces lipolytica*, and *Turolopsis candida* [110].

Dye-degrading enzymes have not yet been well-studied on yeasts. However, it is possible that yeasts also produce putative dye-degrading enzymes similar to filamentous fungi. Some putative oxidative enzymes such as ligninolytic enzymes were found from *Pyricularia oryzae* [111], while some lignin peroxidase activities were detected from the cells of *Saccharomyces cerevisiae* after the decolorization of Methyl Red [112].

### 5.3. Biological Treatment of Dyes Using Algae

Algae are photosynthetic organisms widely distributed in different aquatic habitats. Just like the microorganisms described above, they also exhibit dye degradation capabilities. The algal cell wall contains many functional groups such as carboxy, carbonyl, hydroxy, phosphoryl, and amide groups that play important roles in dye decolorization [113]. Some algae are also able to assimilate the dye chromophores. They possess enzymes that can transform the dyes to H_2_O and CO_2_, leading to the production of algal biomass [75]. 

Microalgae isolates, namely *Chlorella vulgaris*, *Anabaena oryzae*, and *Wollea sacata*, showed efficient decolorization and degradation of different dyes from various dye classes, including the azo dye Orange G [114]. It was also shown that species from *Chlorella* and *Oscillatoria* degrade azo dyes to aromatic amines and further degrade these aromatic amines to simpler organic compounds [115]. Thus, algal biomass can be exploited for the potential treatment of azo dyes, especially as they thrive predominantly on aquatic environments, where textile dye effluents go.

### 5.4. Biological Treatment of Dyes Using Bacteria

Bacteria have been the subject of different studies since they offer several advantages in a biotechnological perspective such as being fast growers, having a plethora of degradative enzymes, and being able to degrade a wide range of dyes. Compared with all discussed microorganism groups, bacterial decolorization has been of considerable interest [22,75]. Although filamentous fungi are effective and potent agents for dye degradation, as discussed above, bacteria are more preferred owing to their faster growth rate and easier handling. The mechanism of dye decolorization and degradation relies on the ability of bacteria to produce enzymes such as azoreductases that can cleave the azo bond (-N = N-) [22,75,116]. Moreover, the ability of bacteria to further reduce or decompose aromatic amines, either aerobically or anaerobically, allows them to have more versatility compared to other organisms [22]. Bacterial decolorization furthermore has more potential for wastewater treatment application, as bacteria are less problematic to handle than filamentous fungi. Some bacterial strains have shown a wide range of substrates that can be reduced. Some have even shown the potential to completely degrade azo dyes. They are also more environmentally friendly and produce less sludge [117,118,119,120,121].

Studies on bacterial decolorization range from pure cultures to mixed bacterial cultures (Table 2 and Table 3). The study of pure cultures in the context of dye degradation allows an in-depth understanding on the mechanism of bacteria toward its behavior against the dyes. It also allows the study of the metabolic and degradation pathways involved [22,122]. Meanwhile, mixed bacterial cultures allow the possibility to explore synergistic activities as well as functional redundancies that can be helpful on dye degradation and may thus exploit alternative ways to degrade dyes [123,124]. Pure cultures can be limited in this sense, as they may encode only for one single activity or pathway to attack azo compounds. In mixed bacterial cultures, it is possible to mix, match, and explore multiple possibilities that would lead to dye degradation. However, the downside is that it is important to identify which bacterial isolates can be mixed and matched to untap the best results. 

**Figure 2:**
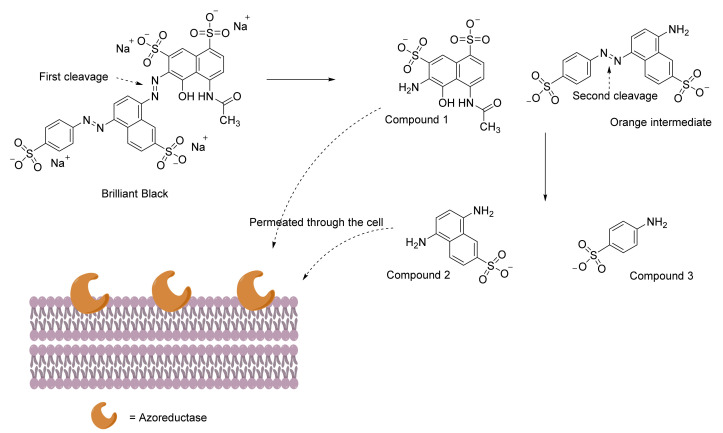
Proposed degradation pathway for Brilliant Black by *Dermacoccus abyssi* MT1.1^T^ while the degradation compounds or intermediates were reported earlier [155].

As the number of studies on dye decolorization and degradation increase, it is also important to investigate the mechanism of how azo dyes are being attacked by these bacteria. Brilliant Black, despite having two azo bridges in its structure, is not reduced simultaneously by Dermacoccus abyssi MT1.1^T^ (Figure 2). Most azoreductases of the organism are known to be localized at the cell membrane. It is discussed that the azo bridge between the two naphthalene rings is cleaved first, producing a naphthol-based compound (compound **1**) and an orange intermediate, 8-amino-5-((4-sulfonatophenyl)diazenyl) naphthalene-2-sulfonic acid. The orange intermediate, which still bears an azo bridge, is attacked by the azoreductase again and produces the second naphthol-based compound (compound **2**) plus sulfanilic acid (compound **3**). Compounds **1** and **2** could not be detected, and therefore, it was proposed that they were translocated into the cell membrane [155].

## 6. Enzymatic Degradation of Azo Dyes

There are several enzymes that have been found to reduce and degrade dyes. Some of the best-known enzymes are manganese peroxidases [156,157,158], lignin peroxidases [103,111,159], laccases [111,160], dye peroxidases [161,162], and azoreductases [163,164,165,166], all of which will be introduced in this section.

Manganese peroxidase is an oxidative enzyme that can destroy phenolic compounds and other xenobiotics with the oxidation of two Mn(II) to Mn(III) [167,168,169]. The Mn(III) compounds are active oxidants, which are typically stabilized by chelating organic acids such as oxalic acid [170]. Manganese peroxidases can break down lignin but can as well decolorize azo dyes and phthalocyanine. It was furthermore demonstrated to degrade the highly recalcitrant polymeric dye, Poly R-478 [171]. Although manganese peroxidase was first discovered in the white rot fungi *Phanaerochaete chrysosporium*, the enzyme was also reported to be responsible for the decolorization of the azo dye Ranocid Fast Blue and the anthraquinone dye Procion Brilliant Blue-H-GR by the bacterium *Serratia marcescens* [172].

Lignin peroxidases are also oxidative enzymes that can degrade lignin, polychlorinated biphenyls, and synthetic dyes [173]. Lignin peroxidases degrade dyes through the oxidation of the phenolic group at the carbon bearing the azo bond to produce a radical group [111]. The water attacks this phenolic carbon and then produces phenyldiazene, which can subsequently be oxidized by a one-electron reaction generating N_2_ [111]. Like manganese peroxidases, this enzyme is often produced by fungal systems. However, there are also bacteria that were reported to have lignin peroxidase activity, such as *Bacillus* sp. strain VUS and *Acinetobacter calcoaceticus* NCIM 2890 [93,174]. *Pseudomonas desmolyticum* NCIM 2112 was also reported to degrade Direct Blue 6 with the involvement of lignin peroxidases, laccases, and tyrosinases [175].

Dye-decolorizing peroxidases (DyP) are heme-peroxidases that were found to have different sequences, structures, and features compared to the classic plant and mammalian peroxidases [176]. These enzymes were discovered to attack azo dyes and the anthraquinone skeleton and hence earned the name dye-decolorizing peroxidases [177]. They contain the highly conserved GXXDG motif in their primary sequences and in addition a conserved Asp, a distal Arg, and a proximal His, which are important for stability, heme-binding, and biocatalysis [162,178,179]. DyPs can be classified into four types (A–D), where types A to C are widespread in bacteria and type D is of fungal origin [180,181]. 

**Figure 3:**
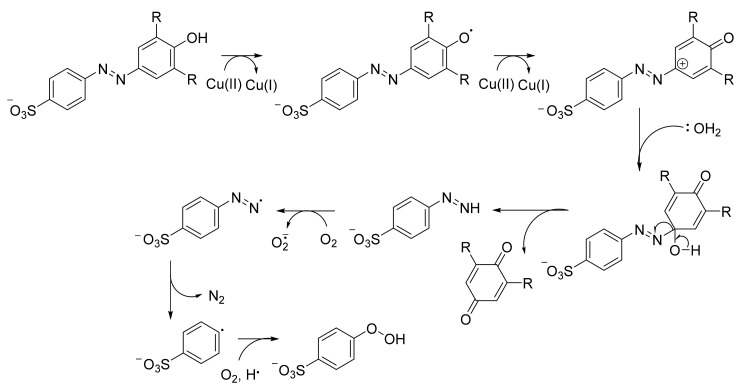
Proposed degradation pathway of azo dyes by laccases. Such activities were proposed for the ascomycete *Pyricularia oryzae* [111].

Laccases, on the other hand, are copper-containing enzymes that can oxidize a wide range of aromatic and inorganic substances [182]. The four Cu^2+^ ions in their active site play an important role in the oxidation of their substrate by taking four electrons from the compound, while the four Cu^2+^ are reduced to Cu^+^ [183] (Figure 3). The reduced laccase transfers the electrons to dioxygen and thereby produces water as it returns to its resting state [183] (Figure 3). Meanwhile, the oxidized substrate automatically decomposes into simple products as it has become an active cation radical [183] (Figure 3). Although some oxidized substrates can revert to the original state, ABTS or 2,2′-azino-bis(3-ethylbenzothiazoline-6-sulfonic acid) can be used as a redox mediator for dye decolorization and degradation [184]. Most laccases have also been discovered from fungi such as *Pichia pastoris* and *Trametes versicolor* [185,186,187]. However, a small number of reports has shown that bacteria can exhibit phenol oxidase activity on azo dyes, as in the case of *Pseudomonas desmolyticum* NCIM 2112 [175].

Although most of the enzymes mentioned are oxidative enzymes, reductive enzymes such as azoreductases are also involved in dye decolorization and degradation. In fact, azoreductases have been a subject of interest for most azo dye decolorization and degradation studies [17,19,22,75,121], thus being worthy of some more in-depth discussion.

## 7. An Insight to Azoreductases

**Figure 4:**
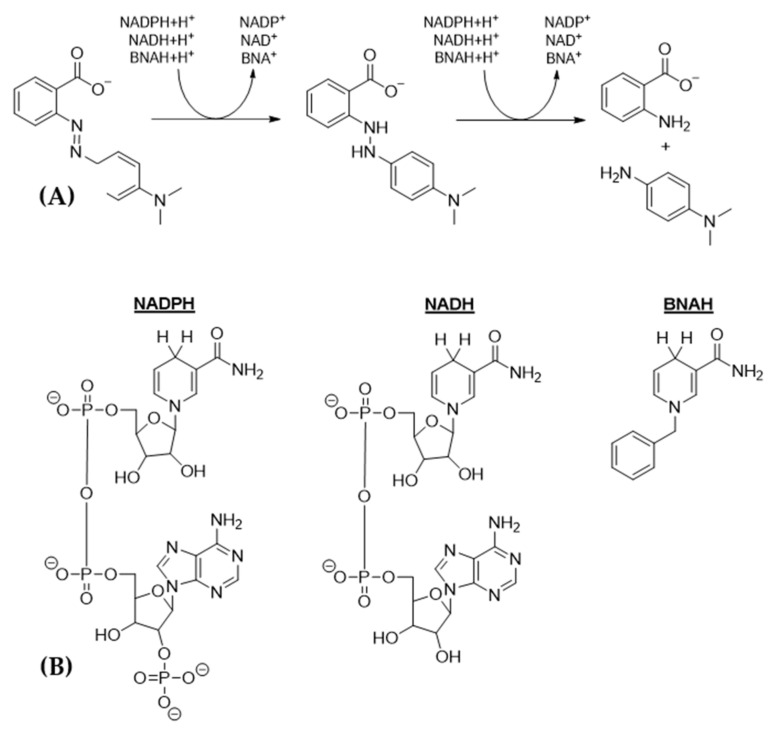
(**A**) Reduction of the model azo dye substrate, Methyl Red, by an arbitrary azoreductase. Most azoreductases use NADPH or NADH as an electron donor to proceed with the reaction. However, some azoreductases can also use an NAD(P)H mimic, such as BNAH, as an electron donor. (**B**) Structure of the electron donors that can be used by azoreductases for the reduction of azo dyes.

Azoreductases are enzymes that cleave azo bonds (-N = N-) present on azo dyes and therefore lead to the formation of colorless aromatic amines [116,122,153]. Most azoreductases operate via a ping-pong bi–bi mechanism (Figure 4). Earlier classifications of azoreductases were based on their prosthetic group. Azorecuctases can depend on flavin mononucleotide (FMN), flavin adenine dinucleotide (FAD), or they can also be flavin-free [188,189,190,191]. Moreover, azoreductases can be further classified based on their preferred co-substrate—NADH, NADPH, or both. However, a recent study by Suzuki (2019) summarized that azoreductases can also be categorized based on sequences [121].

Accounting for the sequence-based classification, clade I azoreductases have the NADPH-binding motif, GXGXXG, which was first observed on AZR from *Bacillus* sp. OY1-2 [164]. The azoreductases that belong to the members of this clade preferentially use NADPH and display about 52–100% sequence identities to each other [121]. Some of the known azoreductases from this group are from *Geobacillus stearothermophilus*, *Rhodobacter sphaeroides*, *Bacillus subtilis* ATCC 6633, and YhdA from *Bacillus subtilis* [117,192,193,194].

Meanwhile, clade II azoreductases do not contain the GXGXXG motif. The primary azoreductase sequences from this clade share about 30–80% identities and prefer NADH over NADPH. Some of the known members are the azoreductases from the human intestinal microflora such as *Enterococcus faecalis* and *Enterococcus faecium* [188,195]. Another member is the YvaB, which was found together with YhdA from *Bacillus subtilis* but lacks the conserved binding motif and in comparison, YvaB and YhdA share only about 26% sequence similarity [121].

Clade III members have about 201 amino acids and share about 25–71% sequence identities [121]. Like clade II members, they lack the NADPH-binding motif, and most have been known as flavoproteins. Some of the known members are azoreductases from *Shewanella oneidensis*, *Rhodococcus opacus*, *Halomonas elongata*, and *Pseudomonas putida* [116,190,196,197,198]. Almost all members of this clade accept both NADH and NADPH. One member can even accept the NADH mimic, 1-benzyl-1,4-dihydronicotinamide (BNAH), as an alternative electron donor. It was shown that AzoRo from *Rhodococcus opacus* 1CP can accept BNAH, which can allow the fast turnover of Methyl Red at pH 7 [198]. Most likely, more members of this enzyme group can accept alternative electron donors, which remains to be demonstrated.

As for clade IV azoreductases, the members do not have the GXGXXG motif but an alternative motif, GXXGXXG, at the N-terminus. Some of the known members were discovered to be flavin-free, such as the azoreductase from *Xenophilus azovorans* [199], *Klebsiella oxytoca* [200], and *Kocuria indica* -DP-K7 [135]. Due to the lack of flavin, the ping-pong bi–bi mechanism does not seem applicable for the azoreductases from this group. It has been stipulated that they catalyze the azo reduction via the formation of a ternary complex where the enzyme binds to NADPH and the substrate transiently [201,202].

Through various studies, it was shown that that several microorganisms possess azoreductases. However, these azoreductases behave differently, as there are no degradation patterns that can be derived even if the enzymes belong to the same clade [121]. The question remains if the azo dye consumptions are just a side activity, as the real physiological role of azoreductases is still to be unraveled as more studies on quinones and azoreductases are completed. 

**Figure 5:**
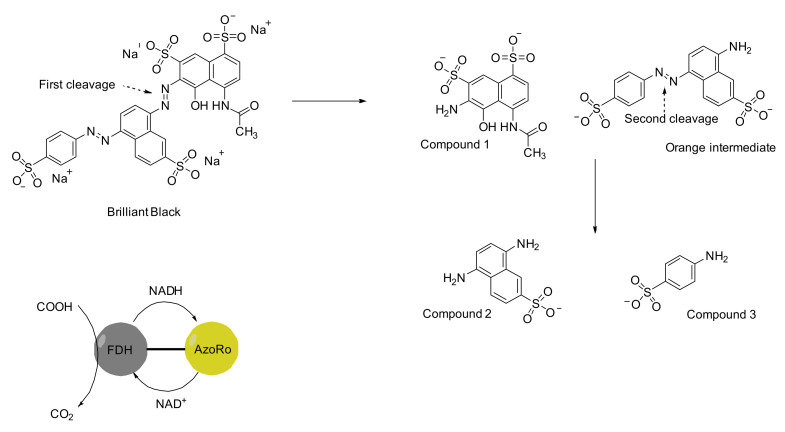
Reduction of Brilliant Black by a fusion protein comprised of the formate dehydrogenase (FDH) from *Candida boidinii* and the azoreductase (AzoRo) from *Rhodococcus opacus* 1CP according to an earlier reported observation of respective degradation compounds or intermediates [203].

Moreover, azoreductases have shown significant versatility in reactivity toward other substrates such as nitroaromatics [121]. Azoreductases have also been a subject of protein engineering. One of the bottlenecks for applying azoreductases is their need of NAD(P)H as an electron donor. NAD(P)H is quite costly and renders the application of azoreductases impractical. However, recent studies have shown that azoreductases can be combined with formate dehydrogenases, which are enzymes that oxidize formate (a cheaper substrate) and donate it to NAD^+^ (Figure 5). Although the fusion protein exhibited only partial degradation of Brilliant Black, this still shows that these enzymes have a potential not only for azo dye degradation but also for other interesting substrates and applications [121].

## 8. Mediators and Varying Energy Sources for a More Efficient Dye Degradation

Azo dye degradation can be improved by the addition of different carbon sources, nitrogen sources, and redox mediators. Some microorganisms can use dyes as a sole carbon source. The combination of a white rot fungus and *Pseudomonas* sp. as a co-culture showed 100% decolorization of Direct Fast Scarlet 4BS without the addition of any carbon source [143]. This was also shown on various actinobacterial isolates and for a *Parabukholderia* sp., where the named organisms were enriched beforehand and isolated by using solely Methyl Red as a carbon source [136]. This was also the case for the bacterium similar to *Hydrogenophaga palleronii*, which was shown to grow on 4-carboxy-4′-sulfoazobenzene [204]. The bacterium was known to degrade sulfanilate and was pre-adapted to the sulfonated azo compound [204]. It was also demonstrated that the microbial community comprised of different bacterial classes can partially degrade azo dyes in the absence of an external carbon source [205], while the bacterial consortium comprised of *Pseudomonas aeruginosa* strain MM01, *Enterobacter* sp. strain MM05, and *Serratia marcescens* strain MM06 could use Reactive Red 120 as a sole carbon source [206]

The addition of carbon sources can also increase the rate of dye decolorization and degradation. This was shown in a study of Saranraj et al., (2018), wherein the decolorization rate of different azo dyes (Reactive Orange 16, Reactive Black B, and Reactive Yellow MR) increased for various isolates, namely *Bacillus odyssey*, *Bacillus thuringiensis*, *Bacillus subtilis*, *Bacillus cereus*, *Alcaligenes* sp., and *Nocardiopsis alba*, when sucrose was added [207]. The addition of 1% glucose also improved the degradation of Brilliant Black BN for different isolates [136]. 

The use of redox mediators can furthermore enhance dye decolorization. Sun et al., (2013) showed that the usage of redox mediators such as anthraquinone-2,6-disulfonate (AQDS), riboflavin, and humic acid increased the decolorization of Congo Red by 394%, 450%, and 258%, respectively [208]. *Halomonas* sp. GYW showed a more efficient decolorization of Acid Red B with the addition of 1,5-dichloroanthraquinone, 1,8-dichloroanthraquinone, anthraquinone, and 1,4,5,8-tetrachloroanthraquinone more than 1.5-fold [209]. Quinones promote electron transfer in different chemical and microbiological reactions [19]. Therefore, the addition of quinones enhances the electron transfer from the electron donor to the electron acceptor, which is often the azo dye. This faster transfer usually leads to an enhanced color removal rate [19,210].

## 9. Prospects on Azo Dye Degradation

Modern solutions to mitigate the repercussions of wastes and pollution are continuously being explored. Azo dyes, which are often used in food, textile, cosmetics, and pharmaceutical industries, pose threats and risks to health and environment. As greener solutions are being considered for the treatment of azo dyes as discussed above, the techniques that can be applied for azo dye degradation also evolve over time. It is important to look through the advancements achieved for azo dye degradation listed below.

### 9.1. Immobilization

Immobilization is a technique of confining enzymes or whole cells into a matrix or onto supports [211]. It is used in different industries especially for the ones that switched to greener solutions. Immobilization allows enzymes or whole cells to be reused and makes them more stable in, e.g., different temperatures or pH. This makes industrial processes more cost-effective and robust. Several supports are available that can range from natural polymers such as alginate, chitin, and sepharose, via synthetic polymers such as Amberlite resins, and to inorganic materials such as zeolites, celites, and silica [211]. There are different ways to facilitate immobilization such as through adsorption, encapsulation, covalent binding, and cross-linking [211].

In the field of azo dye degradation, several studies have already shown the possibility of immobilizing enzymes or whole cells. The study of Chen et al., (2003) exhibited the possibility of immobilizing a microbial consortium with phosphorylated polyvinyl alcohol: immobilized cell beads showed about 75% dye decolorization even with 500 mg/L concentration of Red RBN after 12 h [212]. Another microbial consortium immobilized to polyvinyl alcohol also yielded better decolorization of Direct Fast Scarlet 4BS and could be reused for more than 30 cycles without affecting the dye degradation activity [143]. Although immobilization can be detrimental to the activity of cells or enzymes, the immobilization of *Lysinibacillus* sp. KPB6 in calcium alginate achieved about 98% degradation of Reactive Blue-250 after 48 h as opposed to free cells, which achieved about 95% degradation within 72 h duration [213]. Meanwhile, the enzyme AzoRo, an azoreductase from *Rhodococcus opacus* 1CP, was immobilized to mesoporous silica which showed significant improvement on its stability, exhibiting activity even on incubation at pH 4 for 60 h and showing better storability [197]. The immobilized laccase from *Cyathus bulleri* showed about 90–95% decolorization of simulated dye effluents for up to 20 cycles [214]. Another immobilized laccase of *Weissella viridescens* LB37 on magnetic chitosan nanoparticles showed increased relative activity, which was 2-fold compared to the free enzyme counterpart, and it presented a high removal capacity for Direct Blue 15, Evans Blue, Reactive Black 5, and Acid Red 37 [215].

### 9.2. Bioreactors

Alongside immobilization, specialized bioreactors are often employed in different industrial processes. Bioreactors are vessels or tanks designed to hold free/immobilized whole cells or enzymes for the transformation of substrates to products. The bacterial consortium comprising *Sphingomonas paucimobilis*, *Bacillus* sp. and an unidentified filamentous bacterium was placed in a continuous stirred bed reactor and continuously fed with textile wastewater where the predicted decolorization rate was at 86% [216]. The bacterium *Enterobacter aerogenes* ES014 was also investigated and tested in a batch reactor wherein after treatment, the water quality of the wastewater improved [217]. The possibility of constructing bioreactors using alkalophilic and thermophilic bacterial consortia was also investigated where the color removal efficiency was about 85 to 94% after 192 h [218]. The setup also exhibited a reduction of nitrites and nitrates and that the dye mixture became non-toxic after treatment [217]. In an airlift bioreactor, it was also shown that *Bjerkandera adusta* OBR105 exhibited more than 90% decolorization for Acid Red 114, Acid Blue 62, Acid Black 172, and Reactive Blue 4 after 10–15 h [219]. Another demonstration of the efficiency of dye decolorization in bioreactors was the immobilization of a laccase from *Ganoderma* sp. KU-Alk4 in copper–alginate beads. This immobilized biocatalyst was also applied in an airlift bioreactor for dye removal. Results showed an enhanced stability of the catalyst toward different temperatures, even maintaining its normal activity at 55 °C, and it displayed a decolorization of Indigo Carmine after 14 runs without supplementation [220]. Meanwhile, a rotating disk reactor was also investigated for the treatment of water containing Direct Red-80 and Mordant Blue-9 using immobilized cells of *Phanerochaete chrysosporium*, and it showed more than 90% decolorization efficiencies for individual dyes after 24 h [221]. This highlights the applicability of these bioreactors for wastewater treatment.

### 9.3. Microbial Fuel Cells

Another emerging technology at present is the application of microbial fuel cells (MFC) for different processes. MFCs take advantage of the ability of microorganisms to convert chemical energy (usually from organic matters) to electricity. It has been shown from different studies that electricity can be generated via wastewater treatment with the simultaneous oxidation of different compounds. This technology has been adapted for azo dye decolorization studies. In the study of Liu et al., the possibility of using azo dyes as cathode oxidants was investigated in a cell designed to accept electrons from the respiration of *Klebsiella pneumoniae* L17 in the anode [222]. It was also shown that different dyes can affect the performance of MFCs, as Methyl Orange generated better results than Orange I and Orange II [222]. Industrial wastes can also be used to feed MFCs, as, e.g., brewery waste was used and presented the possibility of reducing Direct Red 80 (200 mg/L) as confirmed by Fourier transform infrared spectroscopy (FT-IR) [223]. In addition, microbial communities that attached to the anode of the setup revealed the presence of proteobacteria, betaproteobacteria, and *Desulfovibrio* [223]. Aside from azo dye decolorization, the removal of sulfides was also observed and coupled with a maximum power output of about 23.5 mW/m in a single chamber air cathode MFC setup [224]. The use of glucose as a substrate to generate electricity and to degrade Acid Navy Blue R was also demonstrated [225]. Different concentrations of dyes were tested, and 200 ppm of dye attained 10.36% Coulombic efficiency and 2236 mW/m^2^ of power density [225].

## 10. Conclusions

Azo dyes have become important in different industries, especially because color plays a huge role in consumer choices. However, with the increased usage of azo dyes, several health and environmental problems have emerged, which are caused by some of these azo dyes and its metabolites. As modern societies are striving toward greener solutions, bioremediation should be taken advantage of. Microorganisms have shown versatile performance not only in the biomedical field but also in the realms of environmental application. Microorganisms have tremendous potential still to be explored, and there are numerous enzymes from various microorganisms that should be further studied. The ability of these microorganisms to accept a broad spectrum of xenobiotics must also be also looked upon. Modern technology has displayed tremendous progress over the past decades. The field of molecular biology and biochemistry has flourished, and the -omics approach is now also being used in different applications in various industries. The combination of these developments and the area of bioremediation, especially in the field of dye degradation, is still an exciting venture to research. Ultimately, these microorganisms can pave the way for a hazard-free conversion of azo dyes and other xenobiotics.

## Figures and Tables

**Table 1:** List of fungal cultures from various decolorization studies and involved enzymes.

Enzyme Class Involved	Culture	Dyes	% Decolorization	References
Laccases	*Marasmius scorodonius*	Congo Red	90%	[101]
		Malachite Green	82%	
		Crystal Violet	69%	
		Methylene Green	63%	
		Reactive Orange 16	48%	
		(+ 1-hydroxybenzotriazole)		
		Remazol Brilliant Blue R	61%	
		(+ 1-hydroxybenzotriazole)		
	*Myceliopthora thermophila*	Acid Blue 74	15.20%	[97]
		Acid Blue 25	53.30%	
		Acid Green 27	67%	
		Reactive Blue 19	31.20%	
		Direct Red 28	9.60%	
	*Trametes versicolor*	Acid Blue 74	88.40%	[97]
		Acid Blue 25	66.00%	
		Acid Green 27	76.00%	
		Reactive Blue 19	64.50%	
		Direct Red 28	11.90%	
	*Aspergillus ochraceus* NCIM 1146	Reactive Navy Blue HER	90.00%	[102]
		Reactive Golden Yellow HER	90.00%	
		Methyl Orange		
			56.00%	
Lignin peroxidases	*Phanerochaete chrysosporium* (Crude lignin peroxidases with 2 mM veratryl alcohol)	Bromophenol Blue	93%	[103]
		Congo Red	54%	
		Methylene Blue	~85%	
		Methyl Green	~85%	
		Methyl Orange	~85%	
		Remazol Brilliant Blue R	~70%	
		Toluidine Blue	80%	
		Poly R-478	46%	
		Poly S-119	80%	
		Poly T-128	48%	
	*Ganoderma lucidum* IBL-05 (with 4 mM veratryl alcohol)	Sandal-fix Red C_4_BLN	66%	[104]
		Sandal-fix Turq Blue GWF	59%	
		Sandal-fix Foron Blue E_2_BLN	52%	
		Sandal-fix Black CKF	40%	
		Sandal-fix Golden Yellow CRL	48%	
	*Bjerkandera adusta* CX-9	Acid Blue 158	~40%	[105]
		Cibacet Brilliant Blue BG	25%	
		Poly R-478	~30%	
		Methyl Green	75%	
		Indigo Carmine	50%	
		Remazol Brilliant Blue R	~90%	
		Remazol Brilliant Violet 5R	<20%	
Manganese peroxidase	*Bjerkandera adusta* CX-9	Acid Blue 158	91%	[105]
		Cibacet Brilliant Blue BG	70%	
		Poly R-478	80%	
		Methyl Green	<20%	
		Indigo Carmine	~45%	
		Remazol Brilliant Blue R	~40%	
		Remazol Brilliant Violet 5R	70%	
	*Cerrena unicolor* BBP6	Congo Red	54%	[106]
		Methyl Orange	78%	
		Remazol Brilliant Blue R	81%	
		Bromophenol Blue	62%	
		Crystal Violet	81%	
		Azure Blue (+gallic acid)	63%	

**Table 2:** List of single bacterial cultures used on different dye decolorization studies.

Culture	Dyes	% Decolorization	References
		(Time of Incubation)	
*Bacillus* sp. AK1	Metanil Yellow	99% (24 h)	[125]
*Sphingomonas paucimobilis*	Methyl Red	99.6% (10 h)	[126]
*Proteus mirabilis*	Red RBN	95% (20 h)	[127]
*Aeromonas hydrophila*	Red RBN	90% (8 days)	[128]
*Brevibacterium* sp. VN-15	Reactive Yellow 107	98% (96 h)	[129]
	Reactive Black 5	95% (144 h)	
	Reactive Red 198	97% (120 h)	
	Direct Blue 71	94% (168 h)	
*Acinetobacter calcoaceticus* NCIM 2890	Amaranth	93% (48 h)	[93]
	Methyl Red	95% (24 h)	
	Amido Black 10 B	87% (72 h)	
	Congo Red	17% (72 h)	
*Bacillus firmus* H4	Novacron Red	80–89% (24 h)	[130]
*Bacillus filamentosus* T13	Novacron Red	80–89% (24 h)	[130]
*Bacillus subterraneus* A36	Novacron Red	80–89% (24 h)	[130]
*Micrococcus luteus* 24M	Congo Red	99% (11 days)	[131]
*Pseudomonas* sp. SUK1	Red BLI	99% (1 h)	[132]
*Pseudomonas* sp. SUK1	Reactive Red 2	>80% (48 h to 72 h)	[133]
*Shewanella putrefaciens*	Acid Red 88	100% (4 h)	[134]
	Direct Red 81	100% (4 h)	
	Reactive Black 5	100% (6 h)	
	Disperse Orange 3	100% (8 h)	
*Kocuria indica* DP-K7	Methyl Red	68% (160 h)	[135]
*Arthrobacter bambusae* DP-A9	Methyl Red	100% (24 h)	[136]
	Brilliant Black	100% (24 h)	
*Leifsonia shinshuensis* DP-L11	Methyl Red	53% (24 h)	[136]
	Brilliant Black	85% (24 h)	
*Dermacoccus nishinomiyaensis* DP-D10	Methyl Red	84% (24 h)	[136]
	Brilliant Black	100% (24 h)	
*Paraburkholderia* sp. DP-P12	Methyl Red	58% (24 h)	[136]
	Brilliant Black	62.5% (24 h)	
*Rhodococcus* sp. UCC 0008	Methyl Red	100% (72 h)	[137]
*Rhodococcus* sp. UCC 0016	Methyl Red	100% (24 h)	[137]
*Staphylococcus* sp. EY-3	Congo Red	>96% (48 h)	[138]
*Kocuria rosea* MTCC 1532	Methyl Orange	100% (72 h)	[139]
*Citrobacter* sp. CK3	Reactive Red 180	95% (36 h)	[140]
*Bacillus* sp. YZU1	Reactive Black 5	95% (120 h)	[141]

**Table 3:** List of microbial consortia used in different dye decolorization studies.

	Culture	Dyes	% Decolorization (Time of Incubation)	References
Bacterial consortium	*Bacillus circulans* BPB8	Textile effluents with mixed azo dyes (Reactive Red, Reactive Brown, Reactive Black) and Cr(VI)	82% (5 days)	[142]
	*Bacillus circulans* HQB947			
	*Bacillis subtilis*			
	*Terribacillus gorriensis*			
Fungal–bacterial consortium	White Rot fungus 8-4* Pseudomonas	Direct Fast Scarlet 4BS (Sole Carbon Source)	100% (30 h)	[143]
Bacterial consortium	*Pseudomonas* sp. ARa	Reactive Red 195 (Maltose and Proteose Peptone)	100% (14 h)	[144]
	*Bacillus* sp. ARc			
	*Bacillus* sp. ARd			
	*Ochrobactrum* sp. ARf			
Bacterial consortium	*Bacillus cereus* BN-7	Acid Red 88	100% (24 h)	[145]
	*Pseudomonas putida* BN-4			
	*Pseudomonas fluorescence* BN-5			
	*Stenotrophomonas acidaminiphila* BN-3			
Fungal–bacterial consortium	*Brevibacillus laterosporus* *Galactomyces geotrichum*	Reactive Red 198	92% (18 h)	[146]
Fungal–bacterial consortium	*Aspergillus ochraceous* NCIM-1146	Rubine GFL	95% (30 h)	[147]
	*Pseudomonas* sp. SUK1	Textile effluent	98% (35 h)	
Bacterial consortium	*Bacillus* sp. AK1	Ponceau 4R	100% (18 h)	[148]
	*Lysinibacillus* sp. AK2			
	*Kerstersia* sp. VKY1			
Bacterial consortium	*Paenibacillus polymyxa*	Reactive Violet 5R	100% (36 h)	[149]
	*Micrococcus luteus*			
	*Micrococcus* sp.			
Bacterial consortium	*Enterobacter dissolvens* AGYP1	Acid Maroon V	93% (20 h)	[150]
	*Pseudomonas aeruginosa* AGYP2			
Bacterial consortium	*Bacillus odyssey* SUK3	Red HE3B	97% (24 h)	[151]
	*Morganella morganii* SUK5			
	*Proteus* sp. SUK7			
Bacterial consortium	*Providencia* sp. SDS	Red HE3B	100% (1 h)	[152]
	*Pseudomonas aeruginosa* BCH			
Bacterial consortium	*Proteus vulgaris* NCIM-2027 (PV)	Scarlet Red Dye Mixture (Scarlet R, Navy Blue HER, Red HE7B, Green HE4BD, Orange HE2R, Navy Blue G, Red HE3B, Navy Blue HE2R, Golden Yellow 24D, Brilliant Blue G, Direct Brown MR, Direct Blue GLL)	100% (3 h)	[153]
	*Micrococcus glutamicus* NCIM-2168 (MG)		88% (72 h)	
Bacterial consortium	*Bacillus subtilis* WGI3	Direct Red 23	70% (48 h)	[154]
	*Bacillus subtilis* WGI4	Direct Yellow 12	84% (48 h)	
	*Bacillus cereus* WGI9	Direct Blue 15	66% (48 h)	
		Dye Mixture	75% (48 h)	
